# Prompt 27-gauge sutureless transconjunctival vitrectomy for bleb-associated endophthalmitis

**DOI:** 10.1007/s10792-017-0747-4

**Published:** 2017-10-23

**Authors:** Bo Xiao, Jin Yang, Yanhua Chu, Quanhong Han

**Affiliations:** 1 Department of Vitreous and Retina, Tianjin Eye Hospital, Tianjin Eye Institute, Tianjin Key Lab of Ophthalmology and Visual Science, Clinical College of Ophthalmology, Tianjin Medical University, 4#, Gansu Road, Heping District, Tianjin, 300020 China; 2 Tianjin Medical University Eye Hospital, Tianjin, China

**Keywords:** Bleb-associated endophthalmitis, 27-Gauge vitrectomy, Transconjunctival, Sutureless

## Abstract

**Purpose:**

To summarize the characters of 6 bleb-associated endophthalmitis (BAE) cases and to report the outcomes of prompt 27-gauge sutureless transconjunctival vitrectomy to treat these cases.

**Methods:**

Retrospective, non-randomized, consecutive case series of patients diagnosed with bleb-associated endophthalmitis who underwent prompt 27-gauge vitrectomy.

**Results:**

The interval to get bleb-associated endophthalmitis from previous surgery was variant from 2 weeks to 36 months. Most of the patients experienced eye pain. The visual acuity was affected quickly. All the patients presented with hypopyon, fibrinous reaction, and vitreous wick. The size of the hypopyon was from 2 to 4 mm. Two patients came with intraocular lenses. Prompt 27-gauge sutureless transconjunctival vitrectomy was performed on all patients with bleb-associated endophthalmitis. None of the patients experienced complications of sutureless vitrectomy such as hypotony or wound leak. The improvement of best corrected visual acuity (BCVA) was significant in four of six patients. The improvement of BCVA was statistically calculated by using logMAR VA (*p* < 0.01). The intraocular pressure (IOP) in all six patients reduced after the surgery. At 6 months’ follow-up, four patients with diffuse blebs had normal IOPs while two patients with cystic or encapsulated blebs had uncontrolled IOPs (> 21 mmHg) and received pressure-lowering agents.

**Conclusions:**

BAE is associated with substantial visual morbidity. Prompt 27-gauge sutureless transconjunctival vitrectomy is an effective and safe way for treating bleb-associated endophthalmitis.

## Introduction

Bled-associated endophthalmitis (BAE) is a rare and severe sight-threatening complication following trabeculectomy. The recent prevalent use of antiproliferative agents such as 5-fluorouracil (5-FU) and mitomycin C (MMC) in trabeculectomy may have increased the incidence of BAE [1–4].

Traditional management of BAE includes intravitreal antibiotic injection and 20-gauge or 23-gauge pars plana vitrectomy (PPV) [1–5]. Here, we report the outcomes of using prompt 27-gauge sutureless transconjunctival vitrectomy to treat BAE, which not only shortens surgical and recovery time but more importantly also minimizes conjunctival damage and better protects filtration bleb as well as creates favorable condition for future glaucoma surgery.

## Cases description

**Fig. 1:**
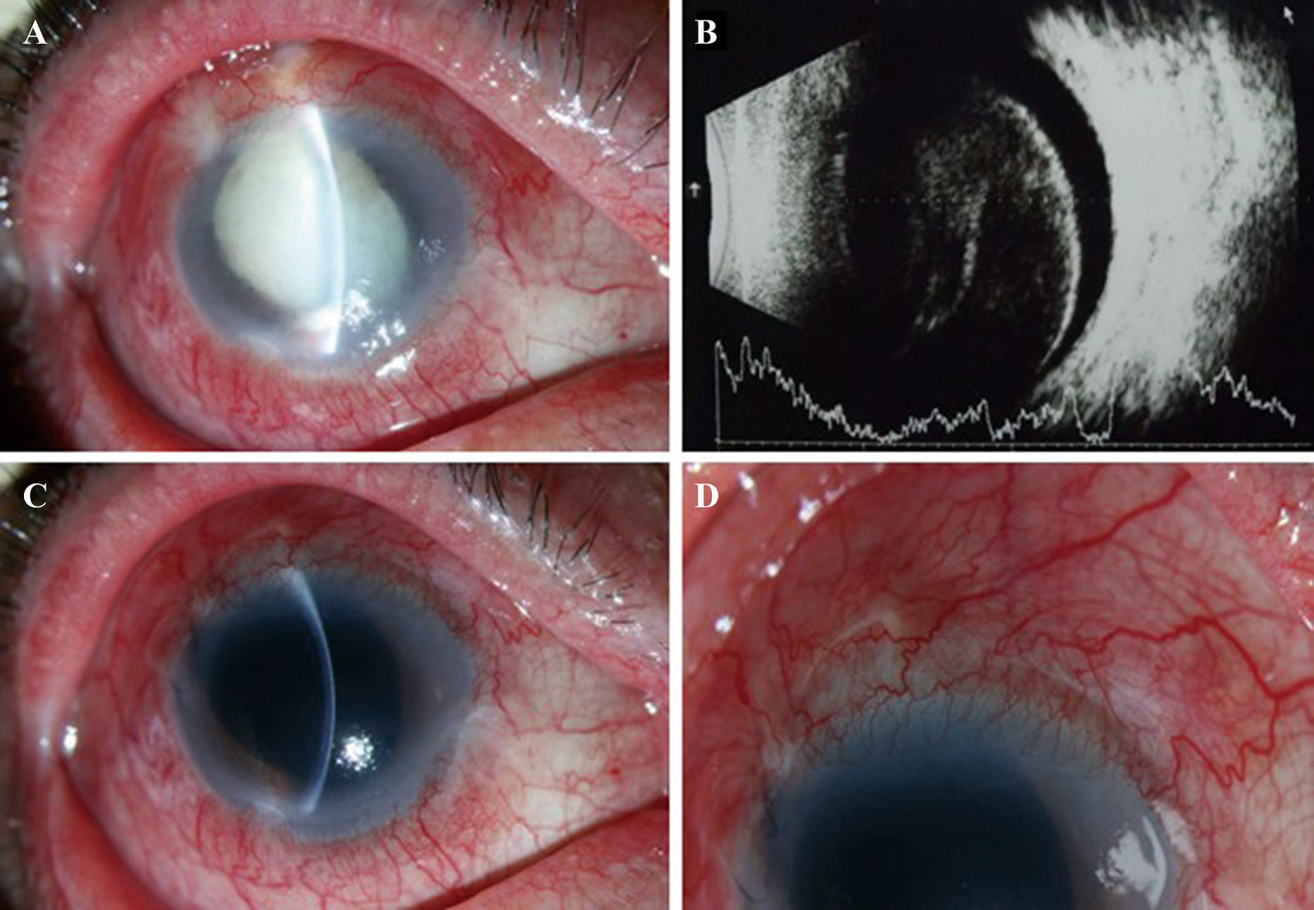
**a** Patient 4’s slit-lamp photograph demonstrating blebitis with mucopurulent infiltrates, hypopyon and exudative membrane in the anterior chamber. **b** Ultrasonography revealing a dense vitreous opacity. **c** Postoperative slit-lamp photograph showing that the hypopyon subsided and the bleb became clear. No subconjunctival hemorrhage was noted. **d** Postoperative slit-lamp photograph showing a functional, shallow, and diffuse bleb

The medical records of six consecutive cases diagnosed with BAE at Tianjin Eye Hospital between December 11, 2014 and February 10, 2017 were reviewed. Their characteristics and clinical features are provided in Table 1 while typical clinical photos of patient 4 are provided in Fig. 1. Cultured organisms were grouped into two categories including: gram-positive coagulase-negative staphylococcus species and gram-positive coagulase positive staphylococcus. Two vitreous culture specimens showed no growth. None of cases had bleb leakage. All patients had the following surgical procedure within 4 h of presentation.

**Table 1:** Characteristics of patients diagnosed with BAE who underwent 27-gauge vitrectomy

Characteristic	Patient 1	Patient 2	Patient 3	Patient 4	Patient 5	Patient 6
Age	51	61	62	65	71	38
Gender	M	M	F	M	F	M
Systemic disease	No	No	No	Hypertension	No	No
Type of glaucoma	PACG	PACG	PACG	PACG	PACG	PAGG
Times of trabeculectomy	1	1	1	2	1	1
MMC	Yes	Yes	Yes	Yes	Yes	Yes
5-FU	No	No	No	Yes	No	No
Bleb site	Superior	Superior	Superior	Superior	Superior	Superior
Bleb leakage	No	No	No	No	No	No
Interval from previous surgery	12 months	7 months	1 month	15 months	2 weeks	36 months
Eye pain	Yes	Yes	Yes	No	Yes	Yes
hypopyon present and size	2 mm	2 mm	3 mm	4 mm	3 mm	4 mm
Surgical wound complication	Fibrinous reaction, vitreous wick	Fibrinous reaction, vitreous wick	Fibrinous reaction, vitreous wick	Fibrinous reaction, vitreous wick	Fibrinous reaction, vitreous wick	Fibrinous reaction, vitreous wick
Culture	Staphylococcus	Staphylococcus	Staphylococcus capitis	No growth	Streptococcus pyogenes	No growth
Time to decrease of vision	12 h	12 h	1 day	4 days	1 day	1 day
Surgery	PPV + Silicon oil	Phaco + PPV + Silicon oil	PPV + air	Phaco + PPV + air	Phaco + PPV + Silicon oil	Phaco + PPV + air
Preoperative BCVA	CF	CF	20/2000	NLP	LP	HM
Postoperative BCVA	20/40	20/60	20/100	NLP	LP	20/100
BCVA at 6 months follow-up	20/25	20/30	20/100	NLP	LP	20/100
Preoperative IOP	31	20	14	15	19	10
Postoperative IOP	26	18	11	8	18	12
IOP at 6 months follow-up	30	20	13	35	20	15
Pressure-lowering agents at 6 months follow-up	2	None	None	2	None	None
Bleb at 6 months follow-up	Cystic	Diffuse	Diffuse	Encapsulated	Diffuse	

*PACG* primary angle-closure glaucoma, *M* male, *F* female, *IOP* intraocular pressure(mmHg), *CF* counting fingers, *LP* light perception, *LP* no light perception, *PPV* pars plana vitrectomy, and *BCVA* best corrected visual acuity

**Fig. 2:**
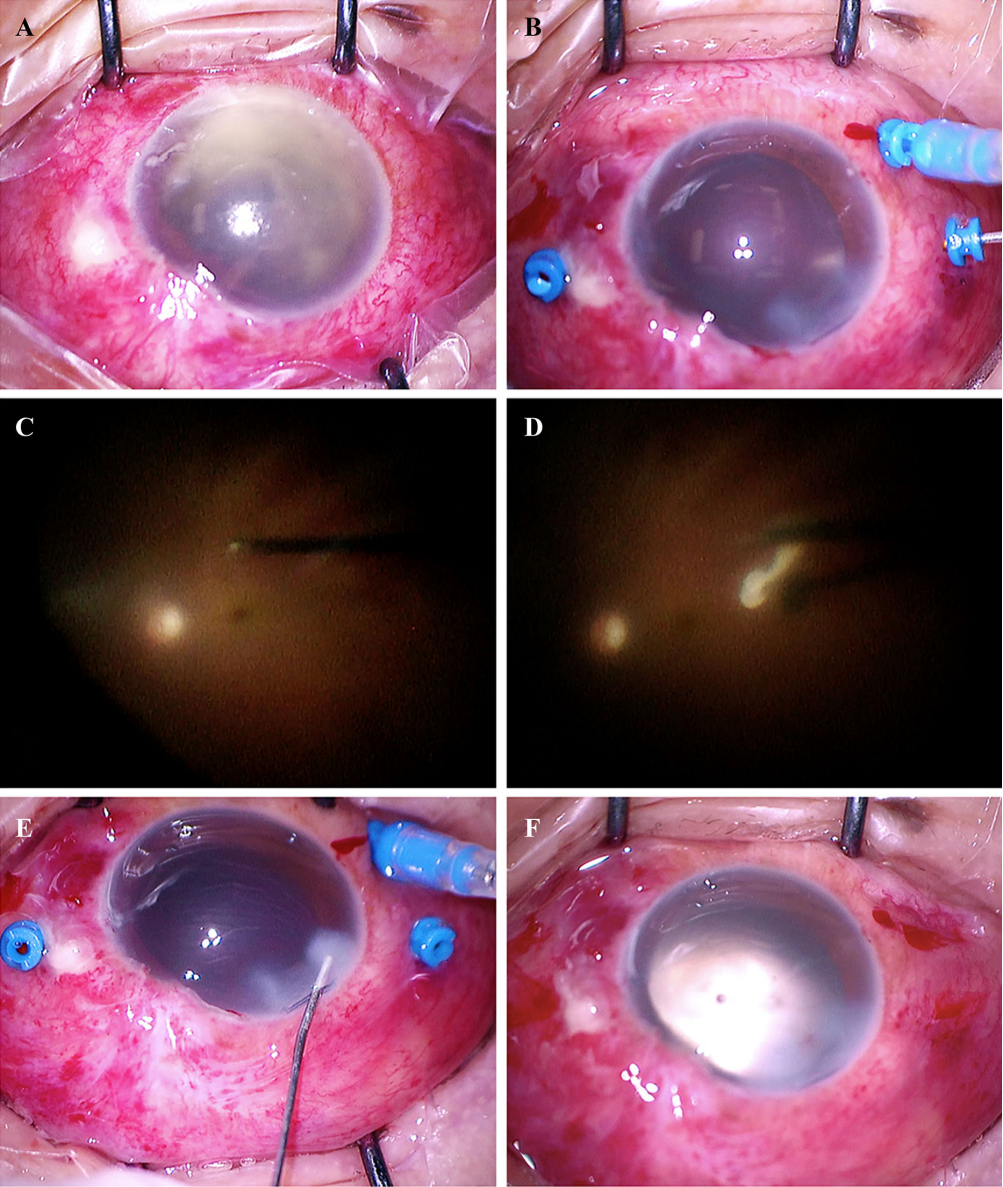
Photographs showing patient 6’s intraoperative findings. A 27-gauge microincision vitrectomy was performed with a wide-angle viewing system allowing for a safe vitrectomy with a clear surgical view. **a** Hypopyon and exudative membrane in the anterior chamber. **b** Carefully created three ports sparing the filtration bleb. Phacoemulsification was performed. **c** Vitreous opacity was removed with a 27-gauge cutter. **d** Internal limiting membrane peeling under the aid of triamcinolone acetonide. **e**, **f** After complete removal of the vitreous opacity, the retina had a close-to-normal appearance. The three ports were successfully closed at the end of 27GMIVS

All patients received retrobulbar anesthesia. Fibrin and inflammatory debris in the anterior chamber were washed out through a corneal incision. The 27-gauge insertion sites were carefully chosen to be as far as practicable from the bleb or scar of prior glaucoma surgery. Operations were performed with the Alcon Accurus Surgical System (Alcon Laboratories, Fort Worth, Texas, USA) and a Binocular Indirect Ophthalmic Microscope (BIOM, Oculus Inc, Wetzlar, Germany) wide-angle viewing system (Fig. 2). The posterior hyaloid was still attached in four cases, and complete posterior vitreous detachment (PVD) was induced. In all cases, marked vitreous reaction, intraretinal hemorrhage, preretinal exudation, and retinal vessel sheaths were noted, and no retinal breaks were found during the operation. Cataract phacoemulsification was performed in three cases. Silicone oil tamponade was performed in two one-eyed cases.

After the surgery, all six patients had mild conjunctival injection with little or no subconjunctival hemorrhage. Five patients had diffuse functional blebs, and one patient had a cystic bleb which seemed to be dysfunctional. No patient experienced complications of sutureless vitrectomy such as hypotony or wound leak.

Statistical analyses for proportional data were done to detect the vision improvement. The improvement of best corrected visual acuity (BCVA) was significant in four of six patients (66.7%, 4 cases) (Table 2). The improvement of BCVA was statistically calculated by using logMAR VA (*p* < 0.01) [6]. The BCVA of patient one and two improved to 20/60 or better postoperatively compared to merely CF preoperatively and further improved to 20/30 or better at 6 months’ follow-up. Patient 3 had an improvement of visual acuity from 20/2000 preoperatively to 20/200 postoperatively. Patient 4 had an improvement of visual acuity from HM to 20/80 at 6 months’ follow-up. The remaining two patients maintained the same vision after the surgery.

**Table 2:** Visual acuity conversion chart for statistic calculation

Patient no.	1		2		3		4		5		6	
Pre/pro-operate	Pre-	Pro-	Pre-	Pro	Pre-	Pro	Pre-	Pro	Pre-	Pro	Pre-	Pro
Measured VA	CF	20/25	CF	20/30	20/2000	20/100	NLP	NLP	LP	LP	HM	20/100
Snellen equivalent	20/2000	20/25	20/2000	20/30	20/2000	20/100	NLP	NLP	LP	LP	20/20,000	20/100
Decimal equivalent	0.01	0.8	0.01	0.67	0.01	0.2	NLP	NLP	LP	LP	0.001	0.2
LogMAR equivalent	2	0.096	2	0.176	2	0.699	NLP	NLP	LP	LP	3	0.699

The intraocular pressure (IOP) in all six cases reduced after PPV was performed. The mean IOP was 19.8 ± 6.7 mmHg (range 14–31 mmHg) preoperatively and 16.2 ± 7.0 mmHg (range 8–26 mmHg) postoperatively, with a mean reduction of 3.6 mmHg. At 6 months’ follow-up, four patients with diffuse blebs had normal IOP while two patients with cystic or encapsulated bleb had uncontrolled IOP (> 21 mmHg) and received two kinds of pressure-lowering agents.

## Discussion

The results of this study showed that BAE is one of the common forms of infectious endophthalmitis. In previous studies, the mean period from initial trabeculectomy to bleb-related infection was from 1 to 16 years [7]. In our study, BAE develops earlier than previous reports. It was from 2 weeks to 3 years. The period difference may come from the amount of investigated patients and individual sanitary habits. The results suggest that careful observation may be required indefinitely after trabeculectomy. Bleb leakage may not be necessarily perceived as a potential risk factor contributing in the development of BAE. Hypopyon was present in all our cases. However, hypopyon absence is noted in previous BAE cases [8]; the significance of this absence must not be underemphasized.

Tan et al. reported of applying 23-gauge transconjunctival sutureless vitrectomy (TSV) for endophthalmitis in six patients, which demonstrated that 23-gauge transconjunctival vitrectomy was safe for both acute and chronic endophthalmitis [5].

In our cases, we demonstrated that 27-gauge transconjunctival sutureless vitrectomy is ideal for BAE cases because it further minimizes conjunctival damage compared to 20-gauge or 23-gauge pars plana vitrectomy. A flexible sclerotomy site could be selected to avoid the bleb or scar of prior glaucoma surgery. Little or no subconjunctival hemorrhage was noted both during the operation and postoperation. This decreased the risk of bleb scarring since blood contains cytokines that are likely to promote bleb fibrosis. Conjunctival tissue which is vital for glaucoma surgery was better protected which created favorable condition for future glaucoma surgery if necessary. All these factors contribute to the low bleb fibrosis rate in our cases. Only one of six patients developed bleb scarring at 6 months’ follow-up. A recently published vitrectomy technique involves modification of surgeons’ position and two sclerotomy sites 45° away from the original position with an infusion cannula inserted inferonasally to avoid damage to the glaucoma drainage implant or filtering bleb. This approach can be considered to apply in the future for a better filtering bleb, IOP maintains and diminishing endophthalmitis incidence [9].

The BCVA in three of the six patients that cultured Staphylococcus were significantly improved compared to preoperative visual acuity. The poor visual outcome of NLP in patient 4 was due to the delayed presentation to the hospital from the onset of symptoms. Patient 5 whose culture resulted in a more virulent bacteria of Streptococcus pyogenes also maintained the same poor vision of LP postoperatively despite the short therapeutic delay. We may thus have concluded that the visual prognosis in BAE patients may be related to the virulence of the infecting bacteria as well as the time elapsed between the onset of symptoms and initial treatment.

In conclusion, our cases demonstrated that prompt 27-gauge sutureless transconjunctival vitrectomy is an effective and safe way to treat patients with BAE, and if diagnosed and treated promptly, good visual acuity and normal IOP may be achieved. The limitation of this paper is that few cases have been summarized. Collaborative multicentric study on the outcome of 27G pars plana vitrectomy in bleb-associated endophthalmitis may provide more cases and robust judgment regarding the superiority of this procedure in the management of this potentially devastating complication of glaucoma filtering surgery.
